# “Train the Trainers” Program to Improve Knowledge, Attitudes and Perceptions About Organ Donation in the European Union and Neighbouring Countries: Pre- and Post- Data Analysis of the EUDONORGAN Project

**DOI:** 10.3389/ti.2023.10878

**Published:** 2023-01-27

**Authors:** Patricia Peralta, Melania Istrate, Chloe Ballesté, Martí Manyalich, Ricard Valero, the EUDONORGAN Consortium

**Affiliations:** ^1^ Surgery and Surgical Specializations Department, Faculty of Medicine, University of Barcelona, Barcelona, Spain; ^2^ European Society of Intensive Care Medicine, Brussels, Belgium; ^3^ Donation and Transplantation Institute (DTI), Barcelona, Spain; ^4^ Department of Anesthesiology, Hospital Clinic of Barcelona, University of Barcelona, Barcelona, Spain; ^5^ Institut d’Investigacions Biomèdiques August Pi i Sunyer (IDIBAPS), Barcelona, Spain; ^6^ Centro de Investigación Biomédica en Red Salud Mental (CIBERSAM), Madrid, Spain

**Keywords:** transplantation, organ donation, training, blended-learning, knowledge

## Abstract

EUDONORGAN, a European Union-funded project to improve organ and tissue donation, included a blended-based “Train the Trainers” program, which was implemented with the support of an international consortium from Croatia, Italy, Slovenia, and Spain. The web-based training included seven modules for which medical aspects, educational tips, and practical activities were scored using a 5-point Likert scale. The overall mean scores of satisfaction were higher than 4 for each module, without significant differences between HCPs and OKPs. In the face-to-face training survey similar scores above 4 were obtained for most items. Knowledge acquisition improved significantly in both HCPs and OKPs, as well as in transplant/donor coordinators, medical doctors, registered nurses, anesthesiologists/intensivists, and intensive care nurses. Improvements in attitudes and perceptions regarding organ donation were also observed, particularly among HCPs. In the accomplishment of the learning process, a successful pass mark of 95% was obtained. The “Train the Trainers” program was associated with an improvement in learning and attitudes of healthcare and non-healthcare professionals for the benefit of organ and tissue donation.

## Graphical Abstract

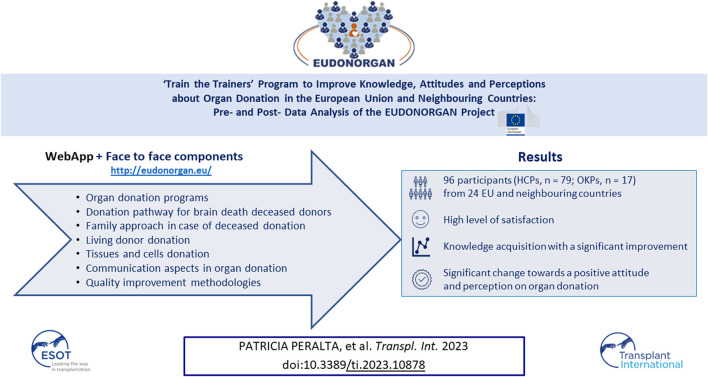

## Introduction

Over the past 50 years, organ transplantation has become an established practice worldwide, bringing immense benefits to hundreds of thousands of patients with end-stage failure of organs for most of whom organ transplantation is the only available treatment (1). The shortage of organs, listed as a major priority, and the supply-demand gap are two limiting factors for organ procurement. In response to these major challenges, the European Commission (EC) issued a communication on organ donation and transplantation (2) that proposed the Action Plan on Organ Donation and Transplantation that complemented the organ specific legislation (3). After a first half-period of completion of the Action Plan, the EC undertook the ACTOR Study, which emphasized the importance of implementing educational activities and improving as there were many opportunities for countries to share experiences and to learn from each other (3). As the study indicated, several EU-funded projects were proposed with the aim of providing training, sharing of knowledge, implementation of programs, development of tools, and to identify the best organizational models (3). In a final assessment of the impact of the Action Plan, a final report (4) provided an overview of the efforts made showing the benefits of the EU-funded resulting in guidelines, trainings, and manuals to exchange knowledge and best practices among countries.

The EU-funded pilot project EUDONORGAN was a pioneer EU-funded project that contributed to the Action Plan as an initiative for increasing organ and tissue donation in the EU and neighbouring countries. To this purpose, two types of core activities focused on training and social awareness were developed and implemented at EU level. The “Train the Trainers” program was based on active learning and adult learning principles and employed a blended learning methodology by means of e-learning (*via* WebApp) and face-to-face training. The course was addressed to healthcare professionals (HCPs) and other relevant key players (OKPs). The objective of this study was to present the results of pre- and post-data analysis of the “Train the Trainers” activities.

## Materials and Methods

### EUDONORGAN Project

EUDONORGAN project was a service contract awarded by the EC from the EU budget, on the initiative of the European Parliament. It was developed by an international consortium, made up of institutions from four European countries, --Croatia, Italy, Slovenia and Spain--, that provided similar organ donation models and successful transplantation rates. The consortium partners were the Institute for Transplantation and Biomedicine-Ministry of Health of Republic of Croatia (Croatia); the Italian National Transplant Centre-Italian National Institute of Health (Italy); the Institute of the Republic of Slovenia for the Transplantation of Organs and Tissues (Slovenia); and the University of Barcelona, Fundació Bosch i Gimpera, the Donation and Transplantation Institute (DTI), and Dinamia, with the support of the Spanish National Transplant Organization (Spain).

The aim of the project was to contribute actively to increase organ donation rates in Europe focusing on two main actions: the implementation of a “Train the Trainers” program on organ and tissue donation, and organizing six social awareness events on organ donation with the support of the trained professionals. Both activities were oriented to HCPs and relevant OKPs, such as patients and patient support groups, representatives of public and governmental agencies, representatives of health institutions, opinion leaders, and the media. EUDONORGAN was launched in September 2016 and lasted 36 months, with the implementation of the “Train the Trainers” program in 2017, and the social awareness events between 2018 and 2019.

The whole timeframe of the project was proposed to be implemented considering the policies established for EU Member States in the field of transplantation and it required to consult and involve the Competent Authorities to establish a European network, following the indications of the Directive 2010/53/EU (1).

### Educational Methodology

#### Training Design, Contents and Participants

The objective of the “Train the Trainers” program was to assist and provided HCPs and relevant OKPs with knowledge, educational strategies and communication techniques to monitor and improve overall performance in the management of donated and transplanted organs. The training included the implementation of a curriculum to support capacity-building efforts and train professionals who will, in turn, be able to conduct future training actions. The design of the program started by establishing a training methodology, the educational contents, and the selection of participants according to the criteria agreed upon by the consortium partners.

The methodology followed analysis of trends in education and literature research to ensure effective educational strategies to engage participants through the “Train the Trainers” program. Based on blended-learning methods that share the common element of engaging participants in doing things and thinking about what they are doing (5), the training offered the advantages of both online (WebApp) and face-to-face components in terms of flexibility of time and place (6,7), accessibility to the best of the educational elements (6), and autonomy with a gradual development of independent learning (7). From a competence-based perspective, blended-learning methods allowed participants to further fine-tune their skills and capabilities, which optimize direct application of experience and knowledge in their own professional environment (8) and promote efficiency, motivation, cognitive effectiveness, and flexibility of learning style (9).

The WebApp (http://eudonorgan.eu) provided a learner-centered platform. Educational modules on organ donation, educational tips and quizzes were delivered through microcapsules of curated content (microlearning) with fine-grained and inter-connected learning activity (10). The storytelling was the narrative learning method used to create a link between lived experience and curricular content (11). Specifically, it showed a family of characters and scenarios through a wide range of game elements in a gradual, entertaining and easy to understand way to keep participants interested and motivated (12).

The face-to-face component employed learning strategies: process mapping exercises, case studies, buzz sessions, collaborative activities and on-ground simulations, that boosted hands-on learning, networking and promoted great interactivity. The methodology followed six adult learning principles (13–16) adapted to the training. This included self-directed experiences; performance-based training to establish a relation between participants’ previous knowledge and their training expectations; experiential learning; critical thinking; learning based on real-world situations; and value learning to further apply the acquired competencies when organizing future training actions on organ donation.

The educational contents were proposed in compliance with the EU legislation (1,17). According to the high-quality standards required (18), these contents should ensure that healthcare personnel directly involved in the chain from donation to transplantation or disposal are suitably qualified or trained and competent, and shall develop specific training programs for such personnel (1) and, consequently, needed to cover the most relevant information on organ and tissue donation. Seven educational modules were designed and adapted to each group of HCPs and OKPs, with the support of international experts, and finally agreed by the members of the consortium. The which included the following content: organ donation programs, donation pathway for brain death deceased donors, family approach in case of deceased donation, living donor donation, tissues and cells donation, communication aspects in organ donation, and quality improvement methodologies. The topics and learning objectives of these modules are described in the Supplementary Table S1.

International experts and participants selection was performed in parallel with the design of the training methodology. Participants from EU Member States and neighbouring countries were invited to join in the training program. The selection of participants followed the recommended criteria agreed by the competent authorities described in the Supplementary Table S2. The objective was to create a heterogeneous pool of trained and dedicated professionals on organ donation that will continue improving in the working environment. Participants were trained on how to best identify donors, how to best organize donation activities (taken into account national specificities) and how to pass on the main positive aspects of donation within the hospitals and to the rest of society (18). The criteria for the selection of HCPs included professionals that were able to demonstrate medical expertise in the field of organ/tissue and cell donation and transplantation. Eligible candidates could be medical doctors (MD) and registered nurses (RN) with different specialties, such as transplant/donor coordinators, anesthesiologists, intensivists, nephrologists, internal medicine physicians, general nurses, or intensive care nurses. The selection of OKPs was focused on actors with proven capacity and motivation to learn and to transfer the knowledge acquired in organ and tissue donation and transplantation *via* the training course, such as active members of patient support groups, communication officers of national and regional authorities, journalists in the field of care, healthcare establishments, and key opinion leaders.

#### Training Implementation

The “Train the Trainers” program started in June 2017 with a series of informative webinars to get all participants familiar with the main topics of the program, the training objectives, and the characteristics of the methodology. Before beginning the training, participants were requested to complete an 18-item test of knowledge and a survey on attitudes and perceptions towards organ and tissue donation. The content of knowledge questionnaires was based on information included in the educational modules. Knowledge questionnaires were different for HCPs and OKPs, whereas the survey on attitudes and perceptions remained the same. Once completed the questionnaires, participants were ready to access to the training program. They were direct responsible for pacing their own self-learning.

The program continued with face-to-face sessions. A total of 9 guests and 11 international experts from six EU countries (Croatia, France, Italy, Slovenia, Spain, and Netherlands) joined the on-site training. The on-site sessions were designed to put into practice the knowledge acquired previously during the online part and to facilitate the switch from the theoretical knowledge to hands-on practice. A learning culture was created with in-class time dedicated to exploring organ and tissue donation topics in greater depth and creating enriching experiences. Apart from the educational contents, an educational kit was provided to participants with essential knowledge on adult learning in medical education and tips on teaching methodologies and strategies.

The training course finished in September 2017. Certificates of achievement were issued and delivered to participants who had completed the program successfully.

#### Evaluation

Continuous evaluation of the participant’s performance was carried out to allow assessing the extent to which the objectives were achieved. The Kirkpatrick impact evaluation model (19) was proposed to measure the educational intervention. The evaluation framework outlined by this author defined four levels of evaluation based on outcomes of satisfaction, learning, change in learner behaviors (20), and organizational change/patient outcome (9). In EUDONORGAN project, this evaluation model was partially used adapted to the design the tailored “Train the Trainers” program and only satisfaction and learning levels based on knowledge, attitudes and perceptions were considered.

The satisfaction level referred to the degree to which learners find the training favorable, engaging, and scientifically relevant (19). After completion of the training, the overall satisfaction of the program was evaluated. For the web-based training, three categories for each educational model, including medical aspects, educational tips, and practical activities were assessed using a 5-point Likert scale (1 = poor, 2 = fair, 3 = good, 4 = very good, 5 = excellent), with a final score as the sum of the scores corresponding to the three categories. For the face-to-face training, 18 items related to different aspects of training methodology and experience, quality of workshops and presentation, specific debates, practical exercises, etc., were defined, and three categories --contents, presentation, and questions and answers--, were assessed for 15 items, whereas other categories were defined for the remaining three items (organization, course information provided, and global evaluation). All items, however, were evaluated using a 5-point Likert scale (1 = poor, 5 = excellent).

The learning level contained three components (knowledge, attitudes and perception (10, 20).

In relation to knowledge acquisition, pre- and post-test tailored-made questions by HCPs and OKPs were designed by three experts. The pre-test multiple-choice questionnaire included 18 items related to the topics given in the seven educational modules, with four possible options, one of which was correct. Only one attempt was allowed. Each item was scored “1” if the answer was correct or “0” if it was incorrect. The post-test multiple-choice questionnaire included 39 items (18 of which were the same questions as those provided in the pre-test). Again, each item was scored “1” if the answer was correct or “0” if it was incorrect. The 18 items that were same in the pre- and post-test were used to assess differences in knowledge acquisition, whereas results of the post-test questionnaires of 39 items were used to establish the accomplishment of training, with a pass mark of 70% of correct responses. The pre-test and post-test multiple-choice questionnaires are reported in the Supplementary Tables S3, S4.

To measure participants’ attitudes pre- and post-surveys were also designed regarding organ and tissue donation. These surveys included a total of seven questions, five of which with three different answer choices and the remaining 2, with different close-ended answers.

Finally, pre- and post-surveys measuring participants’ perceptions of the process of donation after brain death consisted of a set of 20 terms (solidarity, stressful, organized, complicated, positive, painful, opportunity, awkward, correct, strange, dignified, mistrustful, respectful, barbaric, encouraging, dubious, clear, chaotic, easy and discreditable) that from their perspective best describe the process of donation after brain death), five of which should be chosen.

### Statistical Analysis

Categorical data are expressed as frequencies and percentages, and continuous data as mean and standard deviation (SD). In the bivariate analysis, the chi-square test or the Fisher’s exact test were used for the comparison of categorical variables, and the Student’s t*-*test, the Wilcoxon signed-rank test or the Kruskal-Wallis test for the comparison of pre- and post-test quantitative data according to conditions of application. Data for HCPs and OKPs were also stratified by gender, age decades, profession, specialty, and position. Statistical significance was set at *p* ≤ 0.05. All data was analyzed by using the Statistical Package for Social Sciences (SPSS), version 10.0 for Windows.

## Results

### Participants

A total of 96 participants (HCPs, *n* = 79; OKPs, *n* = 17) from 24 EU and neighbouring countries completed the training program. In the group of HCPs, there were 32 men and 47 women, with a mean (SD) age of 40.1 (8.4) years, whereas in the group of OKPs, there were 4 men and 13 women, with a mean age of 40.8 (11.4) years. In the group of HCPs, 51.1% of participants were anesthesiologists or intensivists and 25.3% were RN. Thirty-seven (46.8%) were transplant/donor coordinators. In the group of OKPs, patients’ group representatives accounted for 41.2% of participants followed by communication experts (29.4%). Profession-related characteristics and countries of origin of participants are shown in Tables 1, 2.

**TABLE 1:** Demographic data and characteristics of healthcare professionals.

Variables	N (%)
Total participants	79 (100)
Gender	
Men	32 (40.5)
Women	47 (59.5)
Age, years, mean (SD)	40.1 (8.4)
Profession	
Medical doctor	49 (62.0)
Registered nurse	27 (34.2)
Medical student	2 (2.5)
Healthcare manager	1 (1.3)
Specialty	
Anesthesiology/intensive care	41 (51.1)
General nurse	20 (25.3)
Intensive care nurse	5 (6.3)
Transplant/donor coordinator	3 (3.8)
Nephrology	2 (2.5)
Internal medicine	2 (2.5)
Other	6 (3.8)
Position	
Transplant/donor coordinator	37 (46.8)
Anesthesiologist/intensive care	26 (32.9)
Medical doctor	3 (3.8)
Other	13 (16.5)
Participants per country	
6, France, Italy	12
5, Belgium, Poland	10
4, Estonia, Greece, Lithuania, Spain	16
3, Bosnia & Herzegovina, Bulgaria, Croatia, Cyprus, Hungary, Ireland, Latvia, Malta, Sweden	27
2, Finland, Netherlands, Portugal, Romania, Serbia, Slovenia	12
1, Turkey, Germany	2

Data expressed as frequencies and percentages in parenthesis unless otherwise stated.

**TABLE 2:** Demographic data and characteristics of other relevant key players (non-healthcare professionals).

Variables	N (%)
Total participants	17 (100)
Gender	
Men	4 (23.5)
Women	13 (76.5)
Age, years, mean (SD)	40.8 (11.4)
Profession	
Patients’ group representative	7 (41.1)
Communication expert	5 (29.4)
Journalist	3 (17.6)
Documentalist	1 (5.9)
Other	1 (5.9)
Participants per country	
2, Bulgaria, Ireland, Spain	6
1, Croatia, Cyprus, France, Hungary, Lithuania, Portugal, Romania, Serbia, Slovenia, Slovakia, Sweden	11

Data expressed as frequencies and percentages in parenthesis unless otherwise stated.

### Satisfaction With the Program

For the web-based training considering medical aspects, educational tips, and practical activities of the seven modules, the overall mean (SD) scores of satisfaction were higher than 4 for each module, with 4.4 (0.6) for module 1, 4.5 (0.5) for module 2, 4.5 (0.5) for module 3, 4.5 (0.6) for module 4, 4.4 (0.6) for module 5, 4.4 (0.6) for module 6, and 4.3 (0.7) for module 7, without significant differences between HCPs and OKPs. In the group of HCPs (Table 3), women scored significantly higher than men in modules 3, 5, and 7, but significant differences by age, profession, specialty or position were not found. In the group of OKPs (Table 4), mean scores were also higher than 4 for all modules, but significant differences by gender, age, and profession were not observed.

**TABLE 3:** Satisfaction with the web-based training program among 79 healthcare professionals.

Categories	Participants	Module 1	Module 2	Module 3	Module 4	Module 5	Module 6	Module 7
Gender								
Men	32	4.2 (0.6)	4.3 (0.6)	4.3 (0.6)	4.3 (0.6)	4.0 (0.7)	4.0 (0.7)	4.0 (0.7)
Women	47	4.5 (0.6)	4.6 (0.5)	4.6 (0.5)	4.6 (0.5)	4.6 (0.6)	4.4 (0.6)	4.5 (0.7)
*p*-value		0.098	0.102	**0.017**	0.071	**0.003**	0.221	**0.007**
Age, years								
25–34	16	4.4 (0.5)	4.6 (0.4)	4.5 (0.5)	4.6 (0.4)	4.5 (0.5)	4.4 (0.6)	4.4 (0.6)
35–44	37	4.3 (0.7)	4.5 (0.6)	4.5 (0.5)	4.5 (0.7)	4.4 (0.7)	4.4 (0.7)	4.3 (0.8)
45–54	20	4.5 (0.7)	4.5 (0.6)	4.6 (0.6)	4.5 (0.6)	4.5 (0.7)	4.3 (0.7)	4.4 (0.6)
55–64	6	4.3 (0.6)	4.5 (0.5)	4.6 (0.4)	4.6 (0.3)	4.3 (0.5)	4.4 (0.7)	4.2 (0.7)
*p*-value		0.882	0.258	1.083	0.668	1.324	0.177	0.464
Profession								
Medical doctor	49	4.4 (0.6)	4.5 (0.6)	4.6 (0.5)	4.5 (0.6)	4.5 (0.6)	4.3 (0.7)	4.4 (0.7)
Registered nurse	27	4.3 (0.3)	4.6 (0.6)	4.5 (0.6)	4.5 (0.6)	4.4 (0.7)	4.4 (0.6)	4.3 (0.7)
Medical student	2	4.3 (0.0)	4.7 (0.5)	4.3 (0.0)	4.5 (0.2)	4.3 (0.0)	4.0 (0.5)	4.3 (0.9)
Healthcare manager	1	5.0	5.0	5.0	5.0	5.0	5.0	5.0
*p*-value		0.846	0.644	0.887	0.97	0.672	0.561	0.726
Specialty								
Anesthesiology/intensive care	41	4.4 (0.6)	4.4 (0.6)	4.5 (0.5)	4.5 (0.6)	0.5 (0.7)	4.3 (0.7)	4.4 (0.7)
General nurse	20	4.2 (0.7)	4.5 (0.6)	4.5 (0.5)	4.4 (0.6)	4.3 (0.7)	4.4 (0.6)	4.2 (0.7)
Intensive care nurse	5	4.3 (0.8)	4.5 (0.7)	4.3 (0.7)	4.5 (0.7)	4.7 (0.5)	4.3 (0.9)	3.9 (1.6)
Transplant/donor coordinator	3	4.4 (0.7)	4.7 (0.6)	5.0 (0.0)	4.7 (0.6)	4.9 (0.2)	4.7 (0.6)	4.9 (0.2)
Nephrology	2	4.7 (0.5)	4.7 (0.5)	5.0	5.0	5.0	4.5 (0.7)	4.5 (0.7)
Internal Medicine	2	4.3 (0.9)	4.2 (1.2)	4.7 (0.5)	4.5 (0.7)	4.7 (0.5)	4.3 (0.9)	3.9 (1.6)
Other	6	4.3 (0.5)	4.5 (0.5)	4.5 (0.4)	4.5 (0.4)	4.5 (0.5)	4.3 (0.5)	4.3 (0.5)
*p*-value		0.898	0.937	0.483	0.885	0.498	0.989	0.726
Position								
Transplant/donor coordinator	37	4.5 (0.5)	4.6 (0.5)	4.6 (0.5)	4.6 (0.4)	4.5 (0.6)	4.4 (0.6)	4.4 (0.7)
Anesthesiologist/intensive care	26	4.2 (0.7)	4.4 (0.7)	4.0 (0.6)	4.4 (0.7)	4.4 (0.7)	4.2 (0.7)	4.2 (0.7)
Medical doctor	3	4.5 (0.7)	4.6 (0.6)	4.6 (0.5)	4.5 (0.7)	4.5 (0.7)	4.5 (0.7)	4.4 (0.7)
Other	13	4.2 (0.9)	4.6 (0.4)	4.3 (0.7)	4.6 (0.5)	4.4 (0.5)	4.1 (1.02)	4.8 (0.4)
*p*-value		0.49	0.401	0.447	0.651	0.97	0.756	0.491
Total	79	4.4 (0.6)	4.5 (0.6)	4.5 (0.5)	4.5 (0.5)	4.4 (0.6)	4.3 (0.6)	4.3 (0.7)

Data as mean and standard deviation in parenthesis. Values in bold mean statistical significance.

**TABLE 4:** Satisfaction with the web-based training program among 17 other key players (non-healthcare professionals).

Categories	Participants	Module 1	Module 2	Module 3	Module 4	Module 5	Module 6	Module 7
Gender								
Men	4	4.3 (1.0)	4.6 (1.0)	4.3 (1.0)	4.3 (1.0)	4.3 (1.0)	4.3 (1.0)	4.3 (1.0)
Women	13	4.6 (0.4)	4.8 (0.3)	4.6 (1.0)	4.6 (0.5)	4.7 (0.3)	4.5 (1.0)	4.3 (0.6)
*p*-value		0.589	0.469	0.631	0.589	0.469	0.221	0.772
Age, years								
25–34	6	4.6 (0.3)	4.7 (0.3)	4.7 (0.7)	4.7 (0.4)	4.7 (0.3)	4.4 (0.7)	4.4 (0.7)
35–44	3	4.7 (0.6)	4.8 (0.4)	4.6 (0.5)	4.8 (0.4)	4.8 (0.4)	4.7 (0.3)	4.6 (0.5)
45–54	7	4.3 (0.7)	4.5 (0.8)	4.3 (0.7)	4.1 (0.8)	4.4 (0.7)	4.3 (0.7)	4.1 (0.8)
55–64	1	5.0	5.0	5.0	5.0	5.0	5.0	4.0
*p*-value		0.585	0.742	0.491	0.351	0.723	0.592	0.582
Profession								
Patients’ group representative	7	4.6 (0.5)	4.6 (0.4)	4.4 (0.6)	4.6 (0.5)	4.6 (0.4)	4.5 (0.5)	4.3 (0.5)
Communication expert	5	4.3 (0.8)	4.5 (0.9)	4.4 (0.9)	4.2 (1.0)	4.5 (0.8)	4.3 (0.8)	3.9 (0.9)
Journalist	3	4.8 (0.4)	4.9 (0.2)	4.7 (0.6)	4.8 (0.4)	4.8 (0.4)	4.8 (0.4)	4.9 (0.2)
Documentalist	1	4.3	4.3	5.0	4.7	4.3	3.3	3.3
Other	1	5.0	5.0	4.7	5.0	5.0	4.6	4.7
*p*-value		0.609	0.55	0.847	0.765	0.7	0.486	0.207
Total	17	4.5 (0.5)	4.6 (0.5)	4.5 (0.6)	4.5 (0.6)	4.6 (0.5)	4.4 (0.6)	4.3 (0.7)

Data as mean and standard deviation in parenthesis.

Regarding the face-to-face training survey, data from HCPs and OKPs were gathered, with more than 80 participants who completed the survey in most of the items, and a highest response rate at 85 participants (88.5%). Results of the face-to-face training also showed high scores (above 4) for all items evaluated, except for communication workshop with scores above 3. In the global evaluation, mean (SD) scores of 4.4 (0.8) were obtained for both categories of “applicability to my job” and “overall course assessment” (Table 5).

**TABLE 5:** Satisfaction with the face-to-face training program in all participants.

Items	Participants	Mean (SD)
1. Welcome session		
Contents	81	4.2 (0.9)
Presentation	81	4.2 (0.9)
Questions and answers	81	4.3 (0.9)
2. Project overview and training methodology		
Contents	82	4.4 (0.9)
Presentation	82	4.4 (0.9)
Questions and answers	82	4.4 (0.9)
3. Online training experience		
Contents	82	4.5 (0.8)
Presentation	82	4.6 (0.9)
Questions and answers	81	4.5 (0.9)
4. Living donation		
Contents	84	4.4 (0.9)
Presentation	83	4.4 (0.8)
Questions and answers	84	4.5 (0.8)
5. Deceased donation		
Contents	84	4.6 (0.8)
Presentation	83	4.7 (0.7)
Questions and answers	84	4.7 (0.7)
6. Quality management presentation		
Contents	82	4.3 (0.9)
Presentation	81	4.4 (0.8)
Questions and answers	83	4.3 (0.9)
7. Quality management workshop		
Contents	84	4.2 (0.9)
Presentation	82	4.2 (0.9)
Questions and answers	83	4.3 (0.9)
8. Teaching and learning strategies		
Contents	83	4.1 (0.9)
Presentation	83	4.1 (0.8)
Questions and answers	83	4.3 (0.9)
9. Communication workshop		
Contents	83	3.7 (1.2)
Presentation	84	3.7 (1.1)
Questions and answers	83	3.9 (1.2)
10. Subject specific debates		
Contents	74	4.2 (0.9)
Presentation	74	4.2 (0.9)
Questions and answers	75	4.2 (1.0)
11. Megacase practical exercise		
Contents	84	4.7 (0.8)
Presentation	84	4.7 (0.8)
Questions and answers	84	4.7 (0.8)
12. Communication exercise		
Contents	83	4.0 (1.1)
Presentation	83	4.1 (1.1)
Questions and answers	83	4.1 (1.0)
13. Group work		
Contents	77	4.5 (0.7)
Presentation	75	4.6 (0.7)
Questions and answers	76	4.5 (0.7)
14. Group work presentation		
Contents	59	4.4 (0.7)
Presentation	58	4.5 (0.7)
Questions and answers	59	4.5 (0.7)
15. Wrap up and next steps		
Contents	52	4.6 (0.7)
Presentation	52	4.5 (0.9)
Questions and answers	52	4.6 (0.7)
16. Organization		
Level of organization	85	4.4 (0.9)
Level of teaching	85	4.4 (0.8)
Technical direction	84	4.8 (4.4)
Secretariat	85	4.5 (0.8)
Educational material	85	4.5 (0.7)
Audiovisual support	85	4.3 (0.7)
17. Course information provided		
Before registration	85	4.2 (1-0)
Alter registration	85	4.4 (0.8)
During the course	85	4.5 (0.8)
18. Global evaluation		
Applicability to my job	85	4.4 (0.8)
Overall course assessment	84	4.4 (0.8)

### Knowledge Acquisition

Knowledge acquisition after training showed a statistically significant improvement in both HCPs and OKPs, with mean (SD) percentages of correct responses increasing from 72% (13.4) to 96.2% (5.6) and from 64% (18.3) to 92.8% (7.3), respectively (Table 6). In the group of HCPs, improvement in knowledge acquisition was significant in all age categories, professions, and specialties. Pre- and post-test comparisons were particularly significant for RN vs. MD and intensive care unit nurses vs. general nurses and other specialties (Table 6). Transplant/donor coordinators showed a meaningful improvement (pre-test 71.5% [13.8] vs. post-test 96.7% [5.6], *p* < 0.0001) as well as anesthesiologists and intensivists. In the group of OKPs, statistically significant improvements in knowledge acquisition were observed in women, age segments 25–34 and 45–54 years, patients’ group representatives and communication experts (Table 6). However, between-group differences either in pre-test or post-test results in HCPs or OKPs were not observed.

**TABLE 6:** Learning (knowledge acquisition) scores in all participants.

Variables	Healthcare professionals (HCPs) (*n* = 79)				Other relevant key players (OKPs) (*n* = 17)			
	Participants	Correct answers, %		*p-*value^a^	Participants	Correct answers, %		*p-*value^a^
		Pre-test	Post-test			Pre-test	Post-test	
Gender								
Men	32	71.4 (12)	94.8 (6.9)	<0.001	4	54.1 (32.5)	87.5 (8.3)	0.109
Women	47	72 (14.3)	97 (4.5)	<0.001	13	67 (11.8)	94.4 (6.4)	0.002
*p*-value^b^		0.693	0.67			0.281	0.096	
Age, years								
25–34	16	72.2 (14.8)	95.8 (6.6)	0.001	6	70.4 (5.7)	96.3 (2.9)	0.026
35–44	37	70.7 (14.1)	97.3 (4.6)	<0.001	3	57.4 (12.8)	88.8 (11.1)	0.102
45–54	20	75.4 (12.9)	96.8 (5.6)	<0.001	7	75 (19.5)	95.8 (5.3)	0.042
55–64	6	70.4 (8.5)	92.6 (6.4)	0.027	1	55.5	94.4	
*p*-value^b^		0.688	0.479			0.281		
Profession (HCPs)								
Medical doctor	49	74.1 (12.4)	95.4 (6.5)	<0.0001				
Registered nurse	27	69.8 (14.7)	97.3 (4.2)	<0.0001				
Medical student	2	55.5 (7.9)	100					
Healthcare manager	1	66.6	100					
*p*-value^b^		0.93	0.173					
Profession (OKPs)								
Patients’ group representative					7	69 (15)	94.4 (3.2)	0.027
Communication expert					5	67.7 (17.7)	92.2 (6.3)	0.068
Journalist					3	42.6 (23.1)	85.1 (12.8)	0.109
Documentalist					1	66.6	100	
Other					1	72.2	100	
*p*-value^b^						0.297	0.232	
Specialty								
Anesthesiology/intensive care	41	75 (12.5)	94.8 (6.6)	<0.0001				
General nurse	20	70.8 (15.8)	97.2 (4.2)	<0.0001				
Intensive care nurse	5	62.2 (10.7)	94.4 (5.5)	0.042				
Transplant/donor coordinator	3	77.7	100					
Nephrology	2	55.5	100					
Internal medicine	2	75.0 (11.8)	97.2 (3.9)					
Other	6	65.3 (13.8)	100	0.043				
*p*-value^b^		0.243						
Position								
Transplant/donor coordinator	37	71.5 (13.8)	96.7 (5.6)	<0.0001				
Anesthesiologist/intensive care	26	75 (12.5)	95.3 (6.1)	<0.0001				
Medical doctor	3	72.2 (11.)	96.3 (6.4)					
Other	13	69.4 (13.9)	96.3 (5.5)	0.001				
*p*-value^b^		0.349	0.852					
Total	79	72 (13.4)	96.2 (5.6)	<0.0001	17	64 (18.3)	92.8 (7.3)	<0.0001

^a^ Within-group comparison.

^b^ Between-group comparison.

Finally, in the 39-item questionnaire to assess the accomplishment of the learning process, a successful pass mark of 95% was obtained.

### Attitudes and Perceptions

Attitudes regarding organ and tissue donation in HCPs and OKPs are shown in Table 7.

**TABLE 7:** Attitudes regarding organ and tissue donation in all participants.

Questions	Healthcare professionals			Other relevant key players		
	Pre-test (*n* = 79)	Post-test (n = 64)	*p*-value	Pre-test (*n* = 17)	Post-test (*n* = 13)	*p*-value
Would you donate your organs after death?						
Yes	78 (98.7)	64 (100)	0.321	13 (76.5)	13 (100)	NA
No	0	0		1 (5.9)		
I do not know	1 (1.3)	0		3 (17.6)		
Would you donate the organs of your relatives after death?						
Yes	69 (87.3)	60 (93.7)	<0.0001	16 (94.1)	13 (100)	NA
No	1 (1.3)	0		1 (5.9)		
I do not know	10 (12.7)	4 (6.2)		0		
If you choose “No” or “I do not know” in the previous question, why? (more than one answer is accepted)						
Religious reasons	0	1 (1.6)	NA	0	13 (100)	NA
Lack of trust in the health system	2 (2.5)	1 (1.6)		0		
Not knowing the wish of the deceased	14 (17.8)	4 (6.3)		3 (17.6)		
Ethical reasons	1 (1.3)	0		0		
Fear of body disfiguration	0	0		1 (5.9)		
Other reasons	25 (31.6)	0		4 (23.5)		
Organ and tissue donation should be part of the end of life care						
Yes	75 (94.9)	64 (100)	0.182	13 (76.4)	12 (92.3)	0.689
No	3 (3.8)	0		1 (5.9)	0	
I do not know	1 (1.3)	0		3 (17.6)	1 (7.7)	
When do you consider that it is the most appropriate moment to talk about organ and tissue donation?						
Anytime	29 (36.7)	24 (37.5)	<0.0001	15 (88.2)	7 (53.8)	0.246
When the death of the patient is predictable	28 (35.4)	22 (34.3)		2 (11.8)	2 (15.4)	
After the patient’s death	22 (27.9)	18 (28.1)		0	4 (30.8)	
Do you agree with the admission to the intensive care unit (ICU) of patients with devastating injuries in whom the treatment has deemed futile, for the solely reason of facilitating organ and tissue donation?						
Yes	70 (88.6)	60 (93.7)	0.810	13 (76.5)	12 (92.3)	0.494
No	4 (5.1)	0		2 (11.8)	1 (7.7)	
I do not know	5 (6.3)	4 (6.2)		2 (11.8)	0	
Do you consider appropriate to employ the same resources to maintain a potential brain dead donor as in any other critical patient?						
Yes	75 (94.9)	61 (95.3)	<0.0001	10 (58.9)	12 (92.3)	0.559
No	0	0		3 (17.6)	1 (7.7)	
I do not know	4 (5.1)	3 (4.7)		4 (23.5)	0	

Answers recorded in the post-test survey showed a statistically significant change towards a positive attitude when referring to the willing to donate organs of their relatives both in HCPs and OKPs. Also, 100% of HCPs and OKPs answered “yes” regarding donation of their own organs after death. An improvement in the percentage of participants that considered that organ and tissue donation should be part of the end of life care, both in HCPs and OKPs was also found.

**FIGURE 1:**
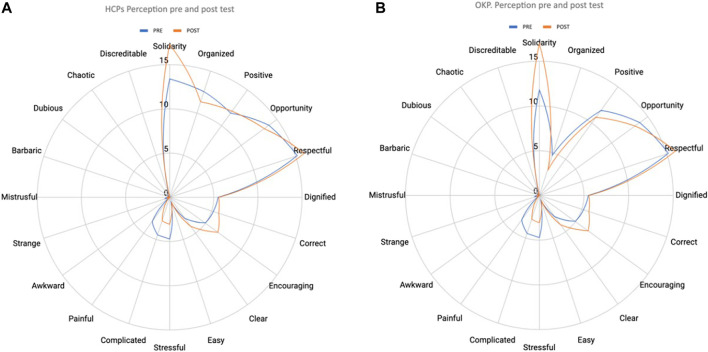
Comparison on perception pre- and post-training in HCPs **(A)** and OKPs **(B)**.

Results of the perception survey showed that both HCPs and OKPs selected more positive than negative terms that better described the process of donation after brain death as compared with pre-test assessment (Figure 1). HCPs significantly improved the selection of solidarity, opportunity, and dignified concepts, and significantly reduced the selection of negative items such as stressful and painful (*p* < 0.05). Positive perceptions were also recorded among OKPs, but differences between pre- and post-test analysis were not statistically significant.

## Discussion

The EUDONORGAN project (21) was proposed within the framework of EU Action Plan on Organ donation and the legislation established in the Directive 2010/53/EU, as one of the initiatives aimed to increase organ availability, to enhance efficiency and accessibility of transplant systems, and to improve quality and safety. The Action Plan advocated appointing of transplant donor coordinators and promoting quality improvement programs in hospitals hence optimizing deceased organ donation, exchanging best practice on donation from living donors, and strengthening communication skills of professionals and patient support groups. Other EU-funded projects focused on improving outcomes from deceased organ donation included to improve collaboration with ICUs (ACCORD) (22), to compare and improve deceased organ donation programs (MODE) (4), to assess protocols and critical steps (COORENOR) (23), and to develop quality system indicators (ODEQUS) (24).

EUDONORGAN was an educational project addressed to HCPs. However, and for the first time in an EU project, OKPs who turned be able to advocate for organ donation and train colleagues in their countries, regions and/or hospitals were also considered to extend the capacity-building efforts to a more heterogeneous group of participants (e.g., patient support groups, journalists, communication experts). Joint involvement of HCPs and OKPs would impact on other aspects, such as standardization of training programmes, and collaboration between countries and sharing of best experiences (4).

As in previous EU-funded training projects, such as ETPOD (25) and EMPODaT (26), the methodology used was blended learning defined in this project as the appropriate mix and use of face-to-face instructional methods and various learning technologies to support planned learning and foster subsequent learning outcomes (27). EUDONORGAN provided an innovative dimension with the use of an instructional delivery method consisting of computer-based training or WebApp with the application of the main adult learning principles in that consider the learner’s role is not only to receive knowledge but also to search, challenge, construct knowledge and change their own perception, views, and beliefs (28). Innovation came by offering game elements, animated characters and scenarios in each of the seven modules on organ and tissue donation following an interactive, enjoyable, and easy to understand manner.

As shown in the satisfaction results, the online educational modules were scored with high values and so it was the methodology used during the face-to-face sessions that boosted hands-on learning, networking, best practice exchange and promoted great interactivity between both groups of participants. They found the training very useful to improve their teaching and communication skills and to organize both trainings and raising awareness events in their daily work context: hospitals, national transplant organizations and/or patients’ associations. Learning results indicated that the training was successfully implemented involving a total of 96 participants from 24 different countries that passed the program with a pass mark of 95%, which is a relevant indicator of a significant increase of knowledge acquisition. These outcomes are even more remarkable in the group of RN as part of the HCPs as professionals active in the field of organ donation and transplantation that resulted as a major factor in maximizing deceased donor potential and eventually increase donation rates (25) and an asset to replicate the training at a national level (28).

Results were also positive in the group of OKPs that become a pool of professionals trained that are part of the entire donation and transplantation chain. In both groups of participants, a change of attitude on their willingness to donate their organs or their relatives was observed. Training also helped improvement towards a positive perception that was noticeable by the increase of positive terms in the post-test. Moreover, both groups could also benefit from further education on various aspects of organ donation and transplantation (4) and on communication skills to support the implementation of public awareness actions and how to communicate with the families of patients, education in schools, generating overall public awareness, and the use of social media (4).

Some limitations of the study should be mentioned. The implementation of “Train the Trainers” program was analyzed, but only at satisfaction and learning levels. The requirements of the EU tender did not foresee the implementation of trainings at local level or regional level, directly related to behavior and result evaluation levels. A post-survey was proposed to optimize the impact of training provided but, the study did not measure the effectiveness of the post-training activities performed by both groups of participants. Assessment of the direct impact of the training program on donation rates was not feasible. However, EUDONORGAN responded very positively to the Action Plan and contributed to promote awareness rising among population with the ultimately improve organ donation rates in the EU and neighbouring countries.

The “Train the Trainers” program was a source of learning and motivation for the professionals. It provided a whole educational framework that allowed a multiplying impact at different levels and types of entities and human supports. The professionals who participated in the study were prepared to organize training actions and events at the local level (university, hospital and/or patient organizations, etc.) and aimed at the target audience. Some of them reported that they had started to implement training actions and a Facebook group was set up in which they continued to interact (https://www.facebook.com/groups/340412829742498/). An evaluation at the clinical and social level could be performed through a follow-up study conducted in European hospitals 2–3 years after the implementation of the training. It would allow to measure whether changes in donation and transplantation occurred in that period.

In summary, organ donation remains a multicomplex process that affects both healthcare professionals and the entire society. Training is a key enabler in healthcare to increase knowledge and skills. This study proves that the methodology used classically in HCPs also applies in OKPs. We identified a significant increase in knowledge and change of attitude and perception that underline the need of permanent education at different levels in relation to organ and tissue donation.

## EUDONORGAN Consortium

Ricard Valero, Melania Istrate (University of Barcelona, Barcelona, Spain); Martí Manyalich, Gloria Páez, Chloe Ballesté (Donation and Transplantation Institute, Barcelona, Spain); Danica Avsec (Institute for Organ and Tissue Transplantation of the Republic of Slovenia, Ljubljana, Slovenia); Mirela Bušić (Institute for Transplantation and Biomedicine, Ministry of Health, Zagreb, Croatia); Paola Di Ciaccio (Centro Nazionale Trapianti, Rome, Italy); Cristina Fernández (Dinamia Consultoría Social, Madrid, Spain).

## Data Availability

The raw data supporting the conclusion of this article will be made available by the authors, without undue reservation.

## Abbreviations

EC, European Commission; EU, European Union; HCPs, healthcare professionals; ICU, intensive care unit; MD, medical doctor; OKPs, other key players; RN, registered nurse; SD, standard deviation.
