# Supplementary material for: “Train the Trainers” Program to Improve Knowledge, Attitudes and Perceptions About Organ Donation in the European Union and Neighbouring Countries: Pre- and Post- Data Analysis of the EUDONORGAN Project
Source: Transpl Int. 2023 Jan 27;36:10878. doi: 10.3389/ti.2023.10878 (PMC9911461; doi:10.3389/ti.2023.10878)
Supplement: Supplementary file 1 [file Table1.DOCX]

**Supplementary Material**

**Table S1**. Educational contents for all participants implemented during the blended-learning program

| **Content** | **Topics** | **Learning objectives** |
| --- | --- | --- |
| Module 1  Organ donation programs | - Living donation - Donation after brain death - Donation after circulatory death (DCD) - Educational tips | - To understand the different types of organ donation programs - To facilitate participants with update information on living donation as a therapeutic alternative, ethical considerations and international recommendations - To gain knowledge on the different types of deceased donation, their main characteristics and world distribution - To gain sound knowledge on the main aspects of uncontrolled and controlled DCD - To gain valuable knowledge on teaching and learning strategies related to the topics of this module |
| Module 2  Donation pathway for brain death deceased donors | - The brain death organ donation process. - The brain death donor critical pathway - Educational tips | - To understand the process of organ donation, the different steps that have to be taken and the actors involved - To know the basic terminology of organ donation and understand the critical pathway - To gain deep knowledge on the steps for deceased organ donation, (identification and referral, brain death diagnosis, donor maintenance, organ recovery, preservation and allocation) the actors involved in each process and the barriers to their correct implementation - To gain valuable knowledge on teaching and learning strategies related to the topics of this module. |
| Module 3  Family approach in case of deceased donation | - Breaking bad news: concepts and communication skills - Family interview for donation - Educational tips | - To get knowledge on communication methodologies required to break bad news - To get familiar with strategies used on how to request the consent for donation - To obtain a complete medical history and detect conditions or behaviors that might imply a risk for the recipient - To gain valuable knowledge on teaching and learning strategies related to the topics of this module |
| Module 4  Living organ donation | - Kidney living donor - Similarities/differences with liver living donor - Educational tips | - To enable participants to learn how to screen a living donor and how to carry out short and long-term follow-up after donation with attention to physical and psycho-social well-being - To identify key factors for protecting the health and safety of living donors - To understand living donation in terms of communication, both to the families and to the broader public - To gain valuable knowledge on teaching and learning strategies related to the topics of this module |
| Module 5  Tissues and cells donation | - Tissue donation - Interlinks with organ donation - Cell donation - Educational tips | - To gain basic knowledge of the tissue and cell donation - To understand the types of tissues and cells that can be donated, their characteristics and selection process of the donors - To know the different uses and applications of donated tissues - To understand the procedures carried out from tissue donation to their transplantation - To understand the interlinks between tissue donation with organ donation - To gain valuable knowledge on teaching and learning strategies related to the topics of this module |
| Module 6  Communication aspects in organ donation | - Contemporary social landscape - Understanding of both the public and the process of behavioral change - Basic principles of communicating with different target groups - Guidelines for strategically approaching communication - How to communicate with mass media and manage adverse publicity - Social media as new communication channels to raise awareness - Educational tips | - To understand public communication, key elements and guidelines - To get familiar with the communication interaction, activities and tools developed at EU level - To acquire sound knowledge on the role of media and social media in social awareness - To learn how to manage adverse publicity - To gain valuable knowledge on teaching and learning strategies related to the topics of this module |
| Module 7  Quality improvement methodologies | - The importance of quality management in health care and organ donation - How to apply quality criteria and indicators in organ donation - Resources already available in quality management and biovigilance - Educational tips | - To understand how quality management applies in organ donation. - To get familiar with methodologies already developed and implemented in EU - To gain valuable knowledge on teaching and learning strategies related to the topics of this module |

DCD: donation after circulatory death, EU: European Union.
