# Supplementary material for: “Train the Trainers” Program to Improve Knowledge, Attitudes and Perceptions About Organ Donation in the European Union and Neighbouring Countries: Pre- and Post- Data Analysis of the EUDONORGAN Project
Source: Transpl Int. 2023 Jan 27;36:10878. doi: 10.3389/ti.2023.10878 (PMC9911461; doi:10.3389/ti.2023.10878)
Supplement: Supplementary file 2 [file Table2.DOCX]

**Supplementary material**

**Table S2**. **Selection of participants**

Participants and experts’ selection was performed in parallel with the design and development of training methodology, teaching activities and resources.

The objective was to perform a mapping of international experts to create a strong community of professionals dedicated to organ donation and ensure the continuity of the project upon its closure. The selection process consisted in several phases:

1. Competent Authorities of Member States and neighbouring countries were provided with the general enrolment criteria and requested to propose up to 7-10 possible participants per country. European patient associations were also contacted with the same request. The participants proposed were requested to provide their resume, motivation letter and letter of support from the sending organization.
2. The applications were carefully analysed, and participants were selected considering the criteria mentioned.
3. The candidates proposed were further discussed with the Competent Authorities where necessary, and once an agreement was reached participants were informed. The selection was completed by June 2017.

The selection followed the recommended criteria agreed by the competent authorities:

- Active in the field
- Achievements in the field (policy changes, better donation rates, studies, and scientific publications in the field, etc.)
- Commitment to actively participate
- Support from sending organization
- Good knowledge of spoken and written English (minimum an intermediate level)
- Previous training in the field (TPM or other) highly recommended but not compulsory
- Experience as a trainer is a strong asset
- Gender equity
- Equitable age and geographical distribution

In specific for HCPs:

- Organ donor/ transplant coordinators
- Intensive care units (ICUs), emergency wards, neurosurgery & neuro-critical care personnel
- Other key donation persons

And the profile of OKPs:

- Patient support groups
- Communication officers of national/regional authorities and of healthcare establishments
- Key opinion leaders
- Journalists in the field of health
