# Supplementary material for: “Train the Trainers” Program to Improve Knowledge, Attitudes and Perceptions About Organ Donation in the European Union and Neighbouring Countries: Pre- and Post- Data Analysis of the EUDONORGAN Project
Source: Transpl Int. 2023 Jan 27;36:10878. doi: 10.3389/ti.2023.10878 (PMC9911461; doi:10.3389/ti.2023.10878)
Supplement: Supplementary file 3 [file Table3.DOCX]

**Supplementary Material**

**Table S3**. Pre- and Post-test Survey with 18 Questions for all Participants

| Topics | Healthcare Professionals | Other Relevant Key Players (Non-Healthcare Professionals) |
| --- | --- | --- |
| Module 1  Organ donation programs | Chose the FALSE statement about deceased donation:   1. Deceased donors refer to any donor that previous to donation has been declared dead by established medical criteria 2. Deceased donors can be divided in two different categories: donors after brain death (DBD) or donors after circulatory death (DCD) 3. DBD is the most frequent type of donation in the world 4. **DCD is the most frequent type of donation in the world** | Which of the following statements about organ donors is TRUE:   1. **There are basically two types of donors: living donors and deceased donors** 2. There are basically two types of donors: brain death donors and cardiac death donors 3. There are basically two types of donors: brain death donors and living donors 4. There are basically two types of donors: cardiac death donors and living donors |
|  | When referring to DCD, chose the TRUE statement:   1. Uncontrolled donors refer to DCD Maastricht type 3 2. Controlled DCD donors can be type 1 or 2 of Maastricht 3. **Maastricht type 3 donors refer to those DCD donors in whom life sustaining treatment limitation has been performed** 4. Type 4 Maastricht donors are the most common type of DCD donors | Organs that human can donate are:   1. **Heart, lung, intestines, pancreas, liver, kidneys** 2. Heart, lung, cornea, pancreas, liver, kidneys 3. Heart valves, lung, cornea, pancreas, stool, kidneys 4. Heart, lung, cornea, osteotendinous tissue, liver, kidneys |
| Module 2  Donation pathway for brain death deceased donors | A possible DBD donor refers to?   1. **A patient with a devastating brain injury or lesion and apparently medically suitable for organ donation** 2. A person whose clinical condition is suspected to fulfil brain death criteria 3. A medically suitable potential donor who has been declared dead based on neurologic criteria as stipulated by the law of the relevant jurisdiction 4. A consented eligible donor in whom an operative incision was made with the intent of organ recovery or from whom at least one organ was recovered for the purpose of transplantation | Mark the statement which is NOT part of the donor coordinator or TPM (Transplant Procurement Manager) role in the donation process:   1. To detect and identify as many donors as possible wherever they are 2. **To diagnose and certify brain death and make sure all the legal requirements are met** 3. To offer the possibility of organ and tissue donation to the relatives of the eligible donors 4. To evaluate the medical suitability of the potential donors in order to avoid the transmission of diseases to the recipient |
|  | These are all absolute contraindications for organ donation, except:   1. Septic shock by methicillin resistant Staphylococcus aureus without antibiotic treatment. 2. HIV Ac + 3. **Advanced age** 4. Unknown cause of death | Who is responsible to detect donors?   1. TPM (Transplant Procurement Manager) 2. Physician and nurse in charge of the patient 3. Medical director 4. **All of them** |
| Module 3  Family approach in case of deceased donation | Concerning the methodology of breaking bad news   1. There is no training required to break bad news 2. It is the personal style of the individual who delivers the bad news that matters, and it cannot be subject to a certain methodology 3. **Health care professionals need to be trained on how to deliver bad news** 4. No communication skills are required | Regarding legal consent systems to express individual consent to donation, the definition of “opting-out system” is:   1. **Everybody is a prospective donor unless they expressed their objection while still alive** 2. Consent to donation has to be obtained explicitly from the donor or an authorised individual (usually the next of kin) 3. All are true 4. All are false |
|  | Regarding legal consent systems to express individual consent to donation, the definition of “opting-in system” is:   1. **Consent to donation has to be obtained explicitly from the donor or an authorised individual (usually the next of kin)** 2. Everybody is a prospective donor unless they expressed their objection while still alive 3. All are true 4. All are false | Which of the following statements best reflects the best practice regarding the interview with the family to evaluate the biological risk of disease transmission?   1. **It should never be omitted, even when we have negative serology results** 2. It can be omitted if we have performed a review of the medical record and we have serology 3. It can be omitted if the family is too distressed to answer and we have serology 4. It can be omitted if the patient does not have any tattoos and is married and we have serology. |
| Module 4  Living organ donation | Which of the following grafts (or their segments) are more frequently transplanted from living donors:   1. Lung 2. Liver 3. **Kidney** 4. Pancreas | All except ONE of these grafts (or their segments) can be transplanted through living donation:   1. Lung 2. Liver 3. Kidney 4. **Heart** |
|  | Living donation with respect to deceased donation (mark the FALSE ONE):   1. Increases the kidney donor pool 2. Increases the number of indications beyond 3. May cause psychological benefits for the donor 4. **May have worse outcomes** | Choose the right statement about living donors:   1. Living donors can only be genetically related to the recipient 2. **Living donors can be genetically and emotionally related to the recipient** 3. Living donors can be genetically and/or emotionally related as well as unrelated to the recipient 4. Everybody can be a living donor |
| Module 5  Tissues and cells donation | Ocular tissue used for transplantation includes (mark the FALSE one):   1. Corneal grafts 2. Anterior and posterior lamellar grafts 3. Scleral tissue 4. **Optical nerve** | How many types of tissue donors are:   1. **Living and deceased donors** 2. Only deceased donors 3. Only living donors 4. Only brain dead donors |
|  | In tissue donation, warm ischemia times (time elapsed from circulatory arrest to tissue removal) should be shorter than (MARK THE FALSE ONE)   1. 12 hours if body was not kept refrigerated 2. 24 h if body was kept refrigerated within 4 hours after 3. **Warm ischemia times for cornea retrieval can be longer** 4. All are correct | Mark the tissue that cannot be donated by living donors:   1. **Ocular tissue** 2. Musculoskeletal tissues 3. Heart valves and vascular segments 4. Human amniotic membrane |
| Module 6  Communication aspects in organ donation | What is the best approach to the public when communicating about organ donation?   1. **To develop a strategic plan and work in multidisciplinary teams** 2. Medical doctors are the best experts for the task 3. Communication with the public is not so important in transplant medicine; if people want, they can find information on the organization’s website, where everything is explained 4. Organizations/national competent authorities need to develop big, national and expensive media campaigns | What is the best approach to the public when communicating about organ donation?   1. **To develop a strategic plan and work in multidisciplinary teams** 2. Medical doctors are the best experts for the task 3. Communication with the public is not so important in transplant medicine; if people want, they can find information on the organization’s website, where everything is explained 4. Organizations/national competent authorities need to develop big, national and expensive media campaigns |
|  | What does the statement “behavioral change is a process” mean in the organ donation context?   1. One well-organized media/communication campaign will linearly and directly result in behavioral change 2. **Over a longer period of time people need several impulses from various contexts to decide to become a donor** 3. When one decides to become a donor, our communication goal has been accomplished for good 4. The public always needs more information and louder media campaigns | What does the statement “behavioral change is a process” mean in the organ donation context?   1. One well-organized media/communication campaign will linearly and directly result in behavioral change 2. **Over a longer period of time people need several impulses from various contexts to decide to become a donor** 3. When one decides to become a donor, our communication goal has been accomplished for good 4. The public always needs more information and louder media campaigns |
| Module 7  Quality improvement methodologies | Which of the following are quality indicator requirements?   1. **Measurable, objective, acceptable, relevant, evidence-based** 2. Specific, measurable, objective, acceptable, relevant 3. Measurable, objective, acceptable, relevant, time-based 4. Specific, measurable, objective, relevant, time-based | Which of the following is the best definition of quality?   1. Is a high level of value or excellence 2. **Is the degree by which the characteristics of a product or service fulfil the objectives for which it was created, where the degree refers to something measurable and fulfillment to the expected outcomes** 3. Is something measurable 4. Is an improvement method |
|  | Quality management in organ procurement at national or regional level considers the analysis of:   1. **Structure, process and outcomes** 2. Structure, quality criteria and indicators 3. Process, outcomes and quality criteria 4. Function, structure, and quality indicators | What is biovigilance NOT useful for?   1. To supervise and assess the risk in order to prevent harm 2. To learn from error 3. **To penalize organ procurement teams in case of error** 4. To improve quality |
| Educational | What is the adult learning process recommended nowadays?   1. Teacher centered. Learners are empty vessels that need to be filled with knowledge, skills and experience. 2. Learner centered. Adult learners are completely independent and exclusively intrinsically motivated. 3. **Mix of appropriate learning strategies (from teacher directed to learner directed)** | |
|  | Does teaching and learning styles matter in the learning process?   1. **Yes. There needs to be a “match” between the learner and the teaching styles used** 2. Maybe, but it is not essential. 3. No. Theorists proved that shifting teaching styles according to the progression of the learning stages does not impact positively the learning process | |
|  | Feedback in the learning process … (chose the FALSE one):   1. Is a difficult part of clinical teaching 2. Is an essential part of medical education 3. **Is judgmental, so experts recommend avoiding it** 4. When constructively given promotes learning and ensures standards are met | |
|  | What are the three interrelated main domains medical education is based on:   1. Transformative education, appreciative education and instrumental learning 2. **Knowledge, skills and attitude** 3. Rote learning, meaningful learning and associative learning 4. Theoretical knowledge, experience and values | |

Correct answers in bold.
