# Supplementary material for: “Train the Trainers” Program to Improve Knowledge, Attitudes and Perceptions About Organ Donation in the European Union and Neighbouring Countries: Pre- and Post- Data Analysis of the EUDONORGAN Project
Source: Transpl Int. 2023 Jan 27;36:10878. doi: 10.3389/ti.2023.10878 (PMC9911461; doi:10.3389/ti.2023.10878)
Supplement: Supplementary file 4 [file Table4.DOCX]

**Supplementary Material**

**Table S4**. Post-test Survey with Questions for all Participants*

| Topics | Healthcare Professionals | Other Relevant Key Players (Non-Healthcare Professionals) |
| --- | --- | --- |
| Module 1  Organ donation programs | Which of the following is FALSE:   1. Nowadays kidney is the most frequently donated organ, followed by liver segments, and occasionally lung lobes, pancreas or intestinal segments. 2. **Many initiatives have been taken to protect vulnerable people from organ commercialization, such as the Toronto declaration** 3. At present, most of the organs removed for transplant come from deceased donors, although in some countries or regions living donors represent a significant number of donation resources 4. The type of relationship allowed for living donation may differ among countries according to their legislation, unrelated donation being the least common one**.** | When referring to Donor after Cardiac Death (DCD):   1. Maastricht type 2 donors refer to persons who arrive dead at the emergency department, after suffering a cardiac arrest 2. Maastricht type 4 donors include those patients who suffer a witnessed cardiac arrest and in whom cardiac resuscitation is started immediately but it is unsuccessful 3. **Maastricht type 3 donors refer to donors in whom the withdrawal of life sustaining treatment is agreed by doctors and family and cardiac arrest is presumed to occur shortly after treatment is withdrawn** 4. Type 1 Maastricht applies to patients where cardiac arrest occurs after brain death has already been diagnosed |
|  | Some of the factors that may impact the profile of donors after brain death (DBD) are:   1. Demographics (older population, older donors) 2. The rate of traffic accidents (more traffic and labor accidents, more DBD secondary to head trauma) 3. The technological advances in neurocritical care 4. **All of them** | Which of the following definitions is TRUE:   1. A living donor is a living human being who donates a certain amount of money to speed up the transplantation process for the ones on the waiting lists 2. **A deceased donor is a human being declared, by established medical criteria, to be dead and from whom cells, tissues or organs are recovered for the purpose of transplantation** 3. A deceased donor is a human being who was diagnosed and declared dead, and in whom organs and tissues are recovered without any type of consent 4. All are correct |
|  | Which statement is TRUE:   1. Warm ischemia damage is not a concerning issue in DCD so preservation techniques or organ recovery can be delayed if necessary 2. **The implementation of DCD programs requires a well-organized structure and excellent coordination of all actors involved in the process** 3. DCD classifications have nothing to do with the occurrence of circulatory arrest before or after treatment limitation 4. There is no critical pathway published for DCD. The existing one concerns only DBD |  |
| Module 2  Donation pathway for brain death deceased donors | Which of the following definitions for brain death donor is INCOMPLETE?   1. Possible deceased organ donor: a patient with a devastating brain injury or lesion and apparently medically suitable for organ donation 2. Potential donor: A person whose clinical condition is suspected to fulfil brain death criteria 3. **Eligible donor: A person who has been declared dead based on neurologic criteria as stipulated by the law of the relevant jurisdiction** 4. Actual donor: A consented eligible donor in whom an operative incision was made with the intent of organ recovery or from whom at least one organ was recovered for the purpose of transplantation | Which are the duties of the organ donor coordinator or TPM (Transplant Procurement Manager):   1. To proficiently coordinate all the steps of the donation process 2. To promote, protect and audit the living donation process and their participating actors 3. To provide information and training on donation and transplantation to different sectors and groups of society, especially to the medical community 4. **All of them** |
|  | Concerning global brain death concept, one of the following statements is TRUE:   1. **Postulates that the irreversible loss of the brainstem function is enough for human death** 2. The most widespread used definition 3. Definition used in some countries such as the UK 4. Used only in some academic settings | The evaluation process (mark the FALSE one):   1. Has the objective to avoid the transmission of infectious diseases and cancer and to ensure that organs will function properly once transplanted 2. Is the responsibility of the TPM (Transplant Procurement Manager) 3. **Should be done only after serology results are known** 4. All of them |
|  | During donor maintenance (mark the FALSE one):  a. Management must be redirected and focused on the support and protection of the organs to be transplanted.  b. Only treatments provided for neurological reasons should be stopped.  c. The goal of ICU care in donor management is to maintain homeostasis  d. **Once the eligible donor is diagnosed brain dead no further treatment is necessary** | These are all absolute contraindications for organ donation, except:   1. **All tumors with no exceptions** 2. HIV Ac + 3. Active acute infections if it was the cause of death or if it affected many organs 4. Unknown cause of death |
|  |  | Organ allocation:   1. Is a complex process interfacing organ recovery and transplantation by matching donated organs with transplant candidates 2. Requires a compromise between equity, justice, efficacy, with the goal to save as many lives as possible and provide transplant recipients with the best possible chance of long-term survival 3. Allocation policies shall not be influenced by favoritism or discrimination based on political influence, national origin, race, sex, religion, socio-economic status or personal / behavioral history 4. **All of them** |
| Module 3  Family approach in case of deceased donation | Which of the following is the best definition of bad news?   1. One that we consider unpleasant or undesirable and would like to avoid delivering 2. One that causes negative emotions on the affected person 3. **One that has a serious and adverse effect on the individual’s view of the future** 4. One that informs about the loss of something important | When is the right time to approach a family for organ donation consent?   1. Before brain death testing in Intensive Care Unit (ICU) 2. After brain death testing in ICU   c. When breaking the bad news of death of the relative  d. **When the family have understood that their relative has died** |
|  | Which one is the recommended request pattern strategy to obtain family consent?   1. Family interview only by ICU physician due to their previous close relationship. 2. Request organ donation as soon as a patient is admitted to the hospital in a very critical condition and bad prognosis due to severe brain injury 3. **Decoupled request with ICU physician informing the brain death diagnosis and transplant coordinator requesting donation** 4. There is not a proper pattern | The recommended way to break bad news is:   1. In a direct manner, right away, so that they can take the news in as soon as possible 2. Only when the person asks for the news through direct questioning 3. **After figuring out what they already know, how many details the family wants to know and making them aware that we are the bearers of bad news** 4. None of them |
|  | The use of metaphors to explain brain death:   1. Should never be used 2. **It is a verbal technique that may facilitate comprehension of such a complicated concept** 3. Should always be used 4. It is an example of reflection of emotions | Which of the following statements is false regarding the family interview for organ donation?   1. **Only half an hour is enough time to carry out a family interview** 2. It is necessary to show empathy and acknowledge their grief 3. Knowledge of verbal and non-verbal communication is necessary 4. The interview time should be adapted to family situation |
| Module 4  Living organ donation | Which is FALSE in reference to the surgical procedure in living kidney donors:   1. **The general principle is that the donor should always be left with his/her worst kidney** 2. When both kidneys are evaluated as equal, the kidney imposing the lowest surgical risk in the recipient will be the chosen one 3. Open or endoscopic nephrectomies may performed 4. The risk of surgical mortality is very low (0.03%) | All, except ONE of the following are advantages of living donation:   1. Grafts may be of better quality from healthy individuals 2. The moment of transplantation can be planned with time 3. **There is no risk for the donor** 4. All are correct |
|  | Which is TRUE in reference to the surgical procedure in liver kidney donors:   1. The general principle is that the donated lobe should always be right one. 2. In cases where the left lobe is used there is an increased risk of the “small-for-size” syndrome in the recipient 3. Mortality rate for living donor hepatectomy is higher to that of the kidney donor 4. **All the above are true** | Informed consent is a necessary step that should be signed (mark the FALSE ONE):   1. **After the evaluation takes place and the donor is considered suitable** 2. To make sure that the donor understands the risks associated with the process 3. Should be given orally and in written form 4. To make sure that the donors understands the long-term consequences for their health |
|  | Living kidney donation (mark the TRUE one):   1. **Has a very low risk of mortality related to donor nephrectomy** 2. In health donors is associated with an increased risk of kidney disease in the long term. 3. Donor age does not influence the risk of donor long term renal disease 4. Living kidney donors have a higher incidence of medical disability and sick leave, as well as a lower life expectancy, than age-matched controls | Cross-over donation:   1. **A living donor who is incompatible with his/her recipient (A) provides a graft to another recipient (B) whose relative´s graft is suitable for the recipient (A)** 2. Is mostly done in lung living donation 3. Is a process in which the living donor gives the graft to the recipient in exchange for the recipient graft that is implanted to the donor 4. It's a form of related-living donation |
| Module 5  Tissues and cells donation | All of the following are specific characteristics of tissue donation that differentiate it from organ donation   1. Higher potential of tissue donation 2. Longer storage time of the retrieved tissues 3. Higher number of potential recipients 4. **All of them** | About deceased tissue donors:   1. Only brain dead donors (DBD) can donate tissues 2. Only cardiocirculatory dead (DCD)donors can donate tissues 3. **Both types of deceased donors can donate tissues** 4. Only DCD donors Maastricht III type can be tissue donors |
|  | Musculoskeletal tissues that can be donated include:   1. Bones 2. Ligaments and tendons 3. Meniscus, cartilage and other soft tissues 4. **All of them** | Tissue donors may be detected:   1. In the Intensive Care Units (ICUs) 2. In other units of the hospitals 3. At morgues and funeral homes 4. **All of them** |
|  | Do to its unique structure human amniotic membrane can be used in all these situation, EXCEPT ONE:   1. **In birth assistance** 2. In arthroplasty 3. In ophthalmology 4. In burns | Medical contraindications for tissue donation:   1. Certain absolute contraindications are similar to those in organ donation 2. Relative contraindications depend of each particular tissue and each particular bank 3. Contraindication of one tissue does not necessarily affect another 4. **All of them** |
| Module 6  Communication aspects in organ donation | What is the most important goal of crisis communication?   1. To promote your organisation and its activities 2. **To maintain trust in and the credibility of your organization and its activities** 3. Not to control the flow of information 4. Not to get involved in a crisis or scandal | What is the most important goal of crisis communication?   1. To promote your organisation and its activities 2. **To maintain trust in and the credibility of your organization and its activities** 3. Not to control the flow of information 4. Not to get involved in a crisis or scandal |
|  | When is European Donation Day celebrated?   1. At annual meetings organised for the media 2. The date is not fixed, the Council of Europe sets the date in case of bad events or scandals 3. It depends when a country celebrates National Organ Donation Day 4. **It is recommended to celebrate it every second Saturday in October** | When is European Donation Day celebrated?   1. At annual meetings organised for the media 2. The date is not fixed, the Council of Europe sets the date in case of bad events or scandals 3. It depends when a country celebrates National Organ Donation Day 4. **It is recommended to celebrate it every second Saturday in October** |
|  | All organizations should incorporate new media in their communication strategy and use it in campaigns   1. Yes, because these days everybody is using social media 2. No, because social media are unsuitable for communicating about organ donation 3. It depends, if you like and know social media, then you should use it 4. **Yes, but carefully and with great caution; some social media can be used as an additional channel for prompt, direct and ongoing communication with the public** | All organizations should incorporate new media in their communication strategy and use it in campaigns   1. Yes, because these days everybody is using social media 2. No, because social media are unsuitable for communicating about organ donation 3. It depends, if you like and know social media, then you should use it 4. **Yes, but carefully and with great caution; some social media can be used as an additional channel for prompt, direct and ongoing communication with the public** |
| Module 7  Quality improvement methodologies | The quality assessment and improvement cycle do NOT include:   1. Plan: identify and prioritize the problems, Analyze the causes and solutions proposed 2. Do: implement the solution agreed 3. Check: evaluate the results 4. **Adopt: define and implement risk measures** | The steps to be considered for quality assessment and improvement are (chose the FALSE one):   1. Problem identification and analysis 2. **Risk management measures** 3. Solution proposal and implementation 4. Result evaluation |
|  | The hospital’s potential of organ donation depends on several factors (chose the FALSE one):   1. Presence of 3rd level trauma services, neurosurgical department, transplant surgery program and ethics committee 2. Number of ICU beds with mechanical ventilation 3. **Number of maternity wards** 4. Attitude of administrative and medical staff towards donation | Quality criteria:   1. Set out the priority areas for marketing and further growth. 2. Are standards of well-being 3. determine the characteristics of a good or service 4. **Are conditions that should be met by the healthcare practice in order to be considered a quality practice** |
|  | Which of the following is NOT a dimension of quality in healthcare?   1. **Social marketing and promotion** 2. Clinical effectiveness 3. Centered on patient 4. Safety | Which of the following statements is FALSE regarding to the definition of indicator?   1. An indicator is a measurement of values 2. An indicator gives an idea of what something is like 3. **An indicator is a punitive system for control** 4. Indicators are expected to indicate and point |

*18 questions are the same as those included in the pre-test (Table S2). Correct answers in bold.
